# Supplementary material for: Development of a p62 biodegrader for autophagy targeted degradation
Source: Nat Commun. 2025 Dec 3;16:10858. doi: 10.1038/s41467-025-65868-9 (PMC12675543; doi:10.1038/s41467-025-65868-9)
Supplement: Supplementary file 1 — Supplementary Information [file 41467_2025_65868_MOESM1_ESM.pdf]

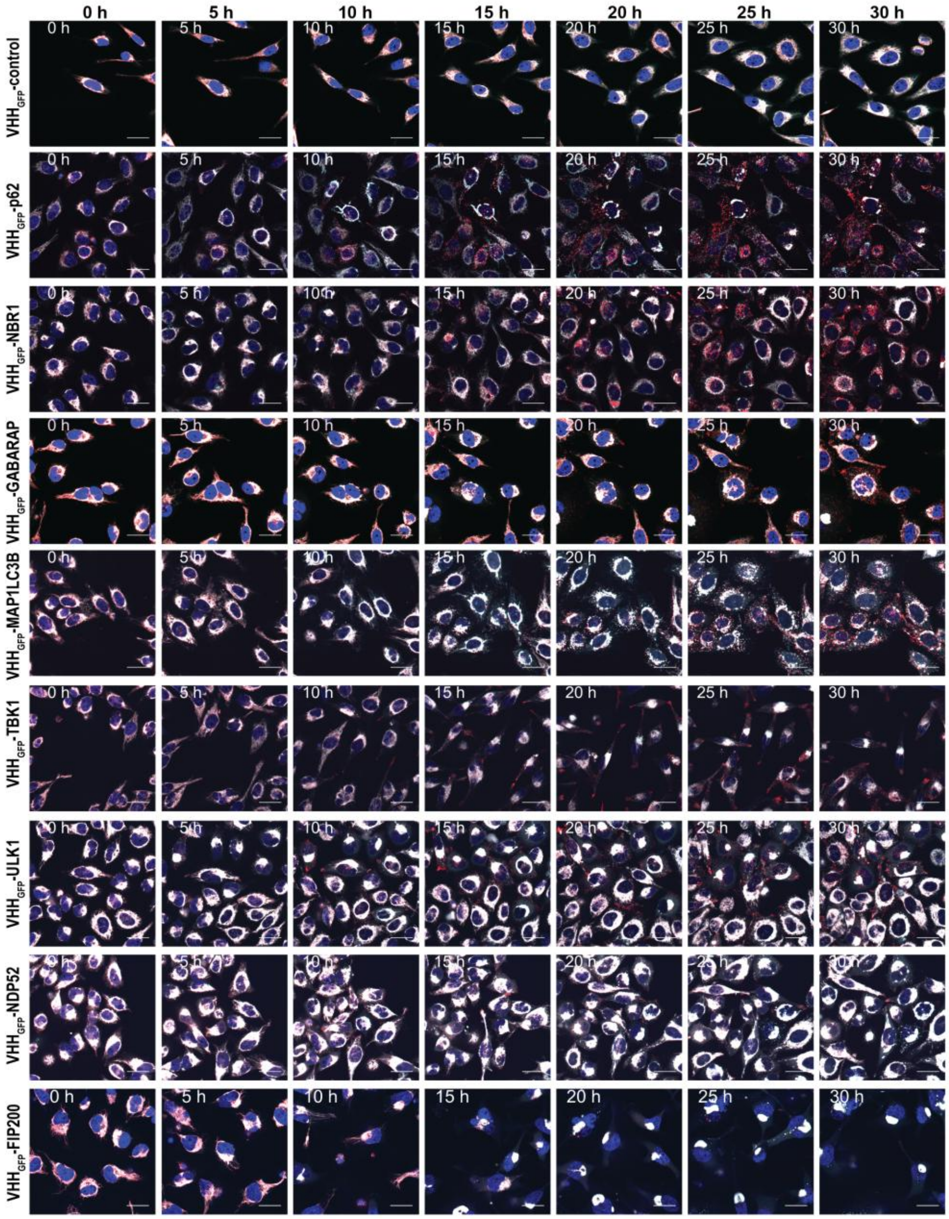

Figure S1. Time-series imaging of stable HeLa mito-mCh-GFP cells with inducible expression of selected fusions of VHH_{GFP} and autophagy effectors.

Expression of the VHH_{GFP} - autophagy effector fusion constructs was induced by addition of 1 µg mL^{-1} doxycycline and the cells were immediately imaged (0h). Fluorescence images of the same field of views were taken every hour for 30 h. Red = mCh, cyan = GFP, blue = nuclei. Scale bars are 30 µm.

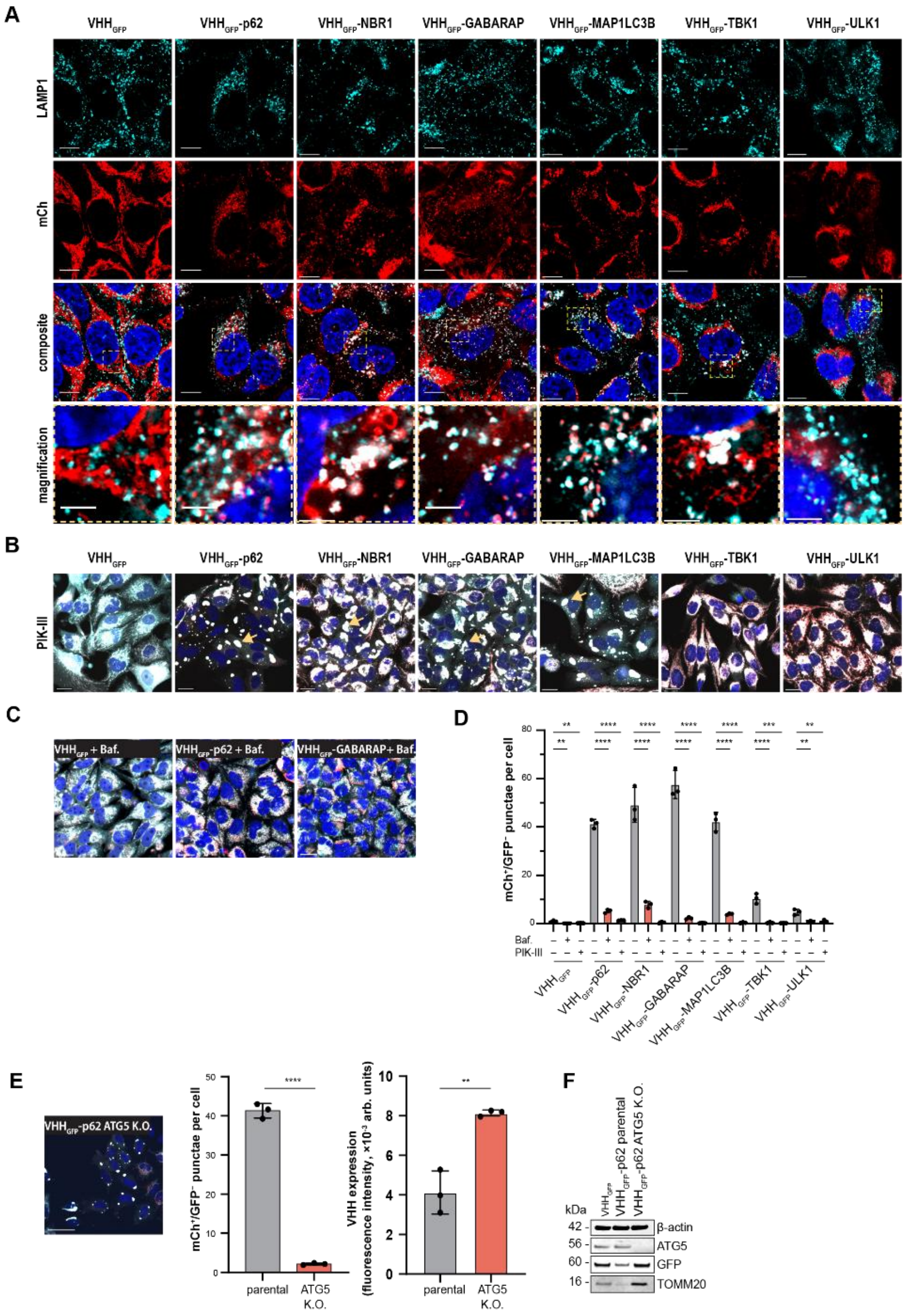

Figure S2. Induced proximity of autophagy effectors results in lysosomal delivery of mitochondria in stable HeLa mito-mCh-GFP cells in an autophagy-dependent manner.

(A) Representative immunofluorescence Airyscan images of stable HeLa mito-mCh-GFP cells with inducible expression of the fusions of VHH_{GFP} and autophagy effectors for 72 h. Cells were fixed and immunostained with anti-LAMP1. Red = mCh, cyan = LAMP1, blue = nuclei. Scale bars are 20 µm and 5 µm (magnification).

(B) Representative confocal fluorescence microscopy images showing stable HeLa mito-mCh-GFP cells with inducible expression of fusions of VHH_{GFP} treated with PIK-III. Red = mCh, cyan = GFP, blue = nuclei. Scale bars are 30 µm.

(C) Representative confocal fluorescence microscopy images showing stable HeLa mito-mCh-GFP cells with inducible expression of fusions of VHH_{GFP} treated with Bafilomycin A1. Red = mCh, cyan = GFP, blue = nuclei. Scale bars are 30 µm.

(D) Quantification of mCh^{+}/GFP^{-} punctae in stable HeLa mito-mCh-GFP cells with inducible expression of the fusions of VHH_{GFP} and autophagy effectors for 72 h in the presence of PIK-III (5 µM) for 72 h or Bafilomycin A1 (100 nM) for 15 h prior to imaging.

(E) Representative confocal fluorescence microscopy image showing stable HeLa mito-mCh-GFP ATG5 K.O. cells with inducible expression of VHH_{GFP} -p62. Quantification of mCh^{+}/GFP^{-} punctae and VHH expression levels in parental and ATG5 K.O. HeLa mito-mCh-GFP cells with inducible expression of VHH_{GFP}-p62 for 72 h. Fluorescence intensities were assessed by immunofluorescence microscopy. Red = mCh, cyan = GFP, blue = nuclei. Scale bars are 30 µm.

(F) Immunoblotting of parental and ATG5 K.O. HeLa mito-mCh-GFP cells with inducible expression of VHH_{GFP}-p62 for 72 h.

Data are shown as mean and standard deviation from n = 3 (panel D and E) independent biological replicates. Statistical analysis was performed using unpaired, two-sided *t*-tests performed on untreated versus treated or parental versus K.O. samples. *P*-value summary: ns = ($P > 0.05$); * = ($P \leq 0.05$); ** = ($P \leq 0.01$); *** = ($P \leq 0.001$); **** = ($P \leq 0.0001$). Exact *p*-values are shown in the source data.

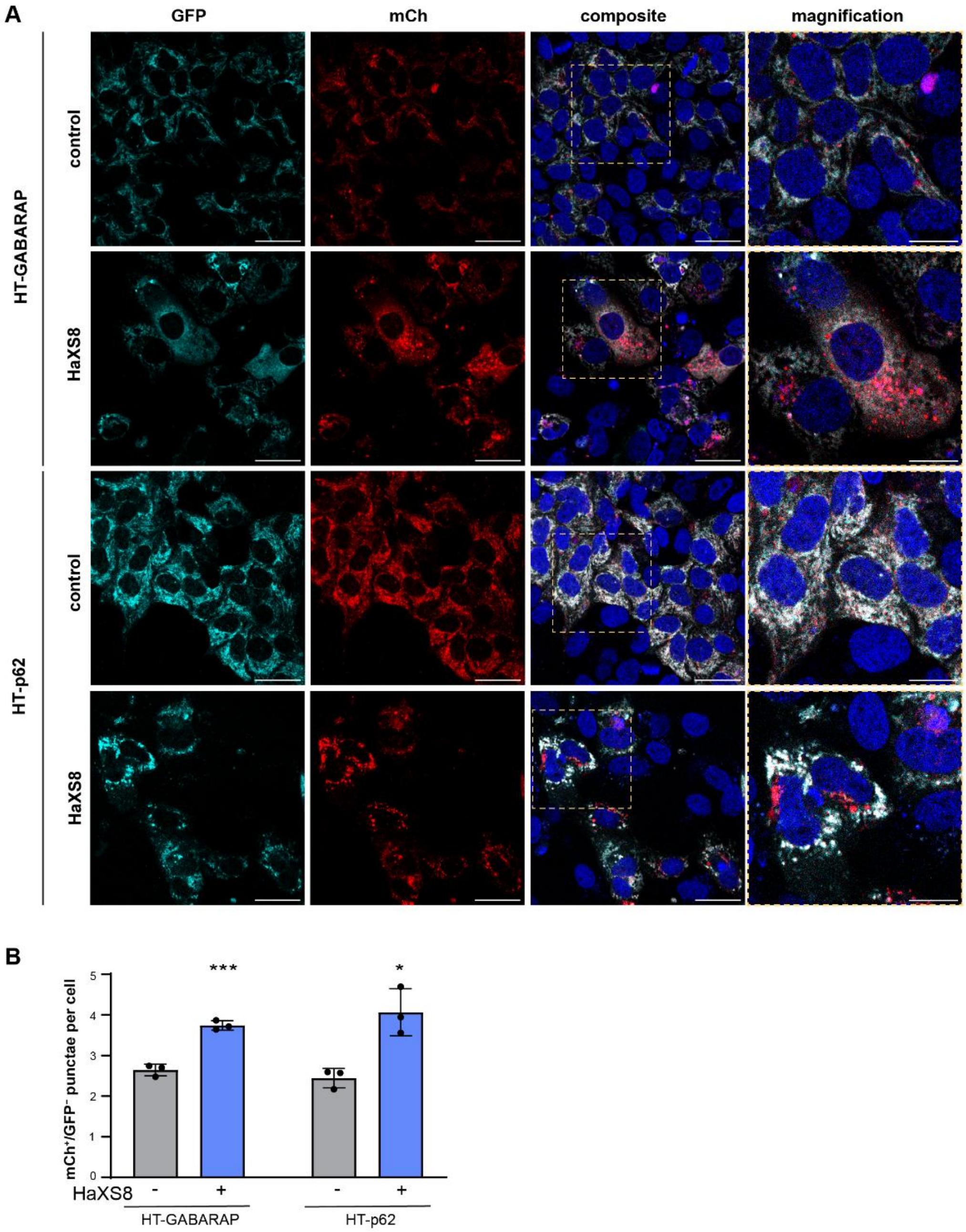

Figure S3. Lysosomal delivery of mitochondria in Huh7 cells using HaXS8.

(A) Confocal fluorescence microscopy images showing live Huh7 mito-mCh-GFP-SNAPtag cells expressing HaloTag-GABARAP or HaloTag-p62. Cells were treated with 100 nM HaXS8 for 72 h. Red = mCh, cyan = GFP, blue = nuclei. Scale bars are 30 µm and 15 µm (magnification).

(B) Quantification of the images displayed in panel (A). The data are shown as mean and standard deviation from n = 3 technical replicates. Statistical analysis was performed using unpaired, two-sided t-tests comparing untreated and treated samples. P-value summary: * = (P < 0.05); *** = (P < 0.001) Exact p-values are shown in the source data.

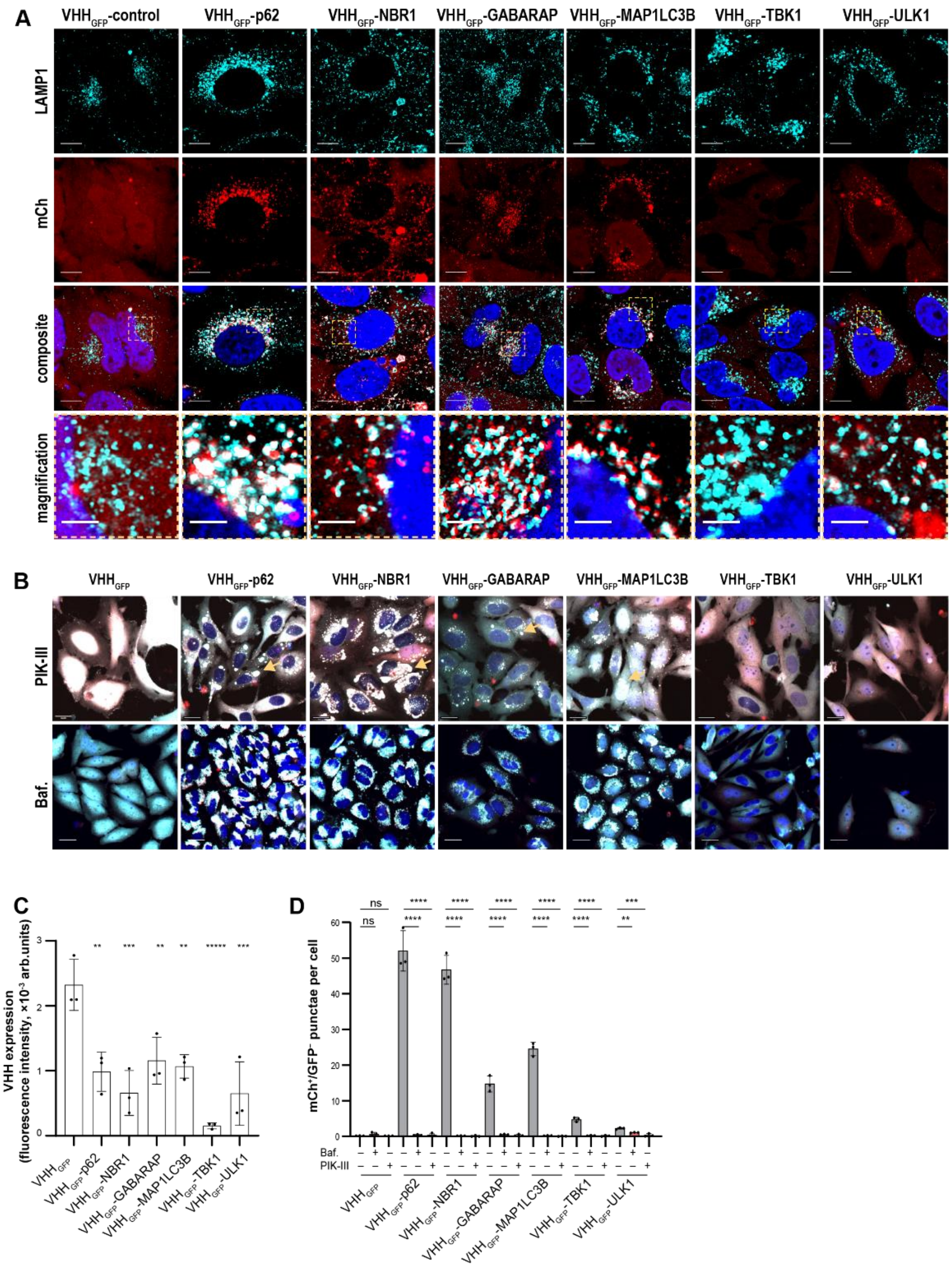

Figure S4. Induced proximity of autophagy effectors results in lysosomal delivery of soluble mCh-GFP in an autophagy-dependent manner.

(A) Representative immunofluorescence Airyscan images of stable HeLa cyt-mCh-GFP cells expressing fusions of VHH_{GFP} and autophagy effectors for 72 h. Cells were fixed and immunostained with anti-LAMP1. Red = mCh, cyan = LAMP1, blue = nuclei. Scale bars are 20 µm and 5 µm (magnification).

(B) Representative confocal fluorescence microscopy images showing stable HeLa cyt-mCh-GFP cells with inducible expression of fusions of VHH_{GFP} treated with PIK-III or Bafilomycin A1. Red = mCh, cyan = GFP, blue = nuclei. Scale bars are 30 µm.

(C) Expression levels of VHH in stable HeLa cyt-mCh-GFP cells 72 h post-induction with doxycycline. Fluorescence intensities were assessed by immunofluorescence microscopy.

(D) Quantification of mCh^+/GFP^- punctae in stable HeLa cyt-mCh-GFP cells with inducible expression of the fusions of VHH_{GFP} and autophagy effectors for 72 h were treated with PIK-III (5 µM) for 72 h or Bafilomycin A1 (100 nM) for 15 h.

Data are shown as mean and standard deviation from n = 3 independent biological replicates. Statistical analysis was performed using an ordinary one-way ANOVA with multiple comparison of each data point against VHH_{GFP} (panel C) unpaired, two-sided t-tests performed on untreated versus treated samples (panel D). P-value summary: ns = (P > 0.05); * = (P ≤ 0.05); ** = (P ≤ 0.01); *** = (P ≤ 0.001); **** = (P ≤ 0.0001). Exact p-values are shown in the source data.

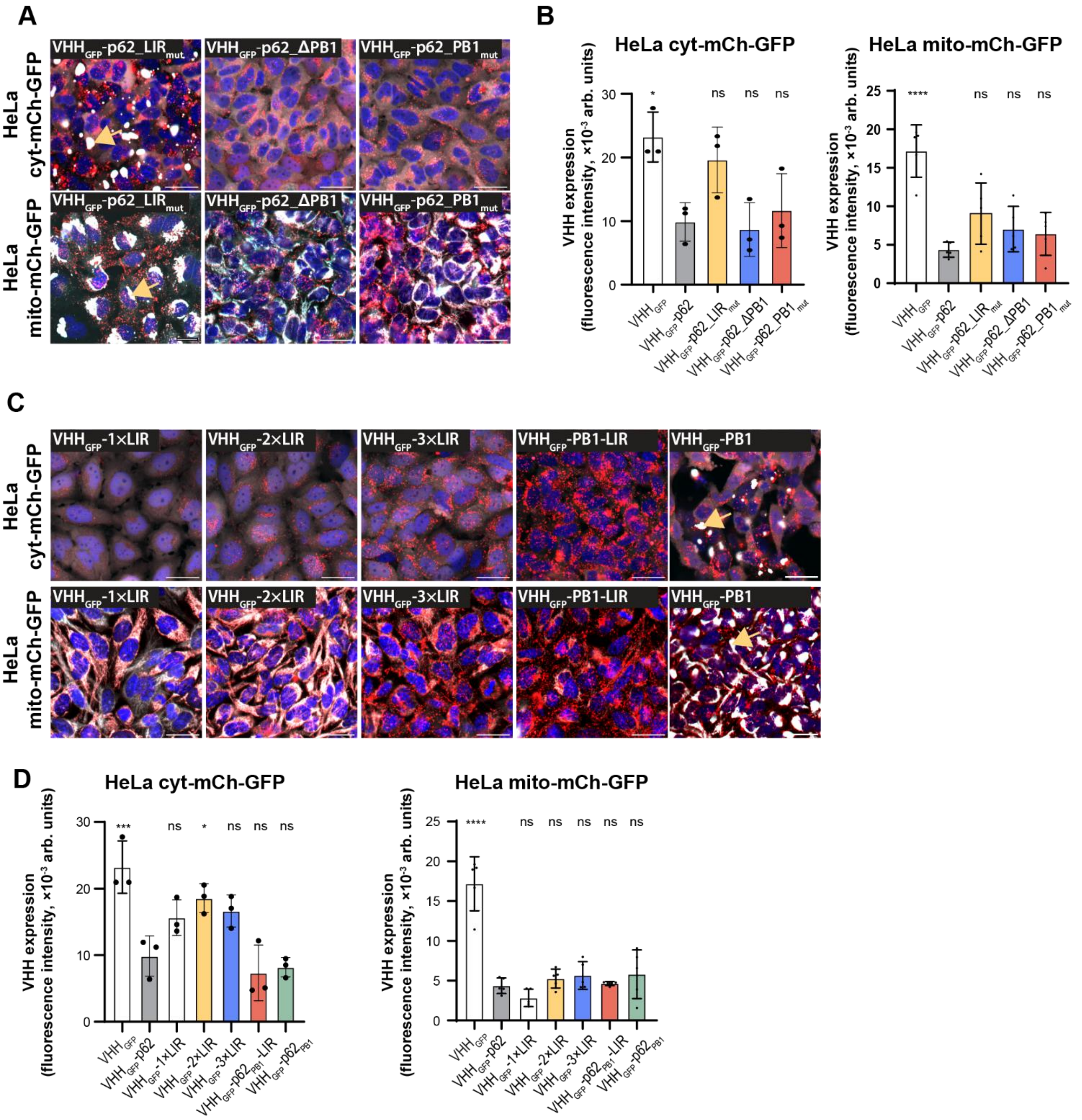

Figure S5. HeLa cyt-mCh-GFP and mito-mCh-GFP with inducible expression of fusions of VHH_{GFP} and p62 mutants.

(A) Representative confocal fluorescence microscopy images showing stable HeLa cyt-mCh-GFP and HeLa mito-mCh-GFP cells with inducible expression of fusions of VHH_{GFP} and p62 mutants for 72 h. Red = mCh, cyan = GFP, blue = nuclei. Scale bars are 30 µm. Large mCh$^+$/GFP$^+$ structures are indicated by yellow arrows.

(B) Expression levels of VHH_{GFP} fusions of panel A. Fluorescence intensities were assessed by immunofluorescence microscopy.

(C) Representative confocal fluorescence microscopy images showing stable HeLa cyt-mCh-GFP and stable HeLa mito-mCh-GFP cells with inducible expression of VHH_{GFP} fusions with LIR and PB1 containing constructs for 72 h. Red = mCh, cyan = GFP, blue = nuclei. Scale bars are 30 µm. Large mCh$^+$/GFP$^+$ structures are indicated by yellow arrows.

(D) Expression levels of VHH_{GFP} fusions of panel C. Fluorescence intensities were assessed by immunofluorescence microscopy.

Data are shown as mean and standard deviation from n = 3 (cyt-mCh-GFP) or n = 5 (mito-mCh-GFP) independent biological replicates. Statistical analysis was performed using an ordinary one-way ANOVA with multiple comparison of each data point against VHH_{GFP}-p62. P-value summary: ns = ($P > 0.05$); * = ($P \le 0.05$); ** = ($P \le 0.01$); *** = ($P \le 0.001$); **** = ($P \le 0.0001$). Exact p-values are shown in the source data.

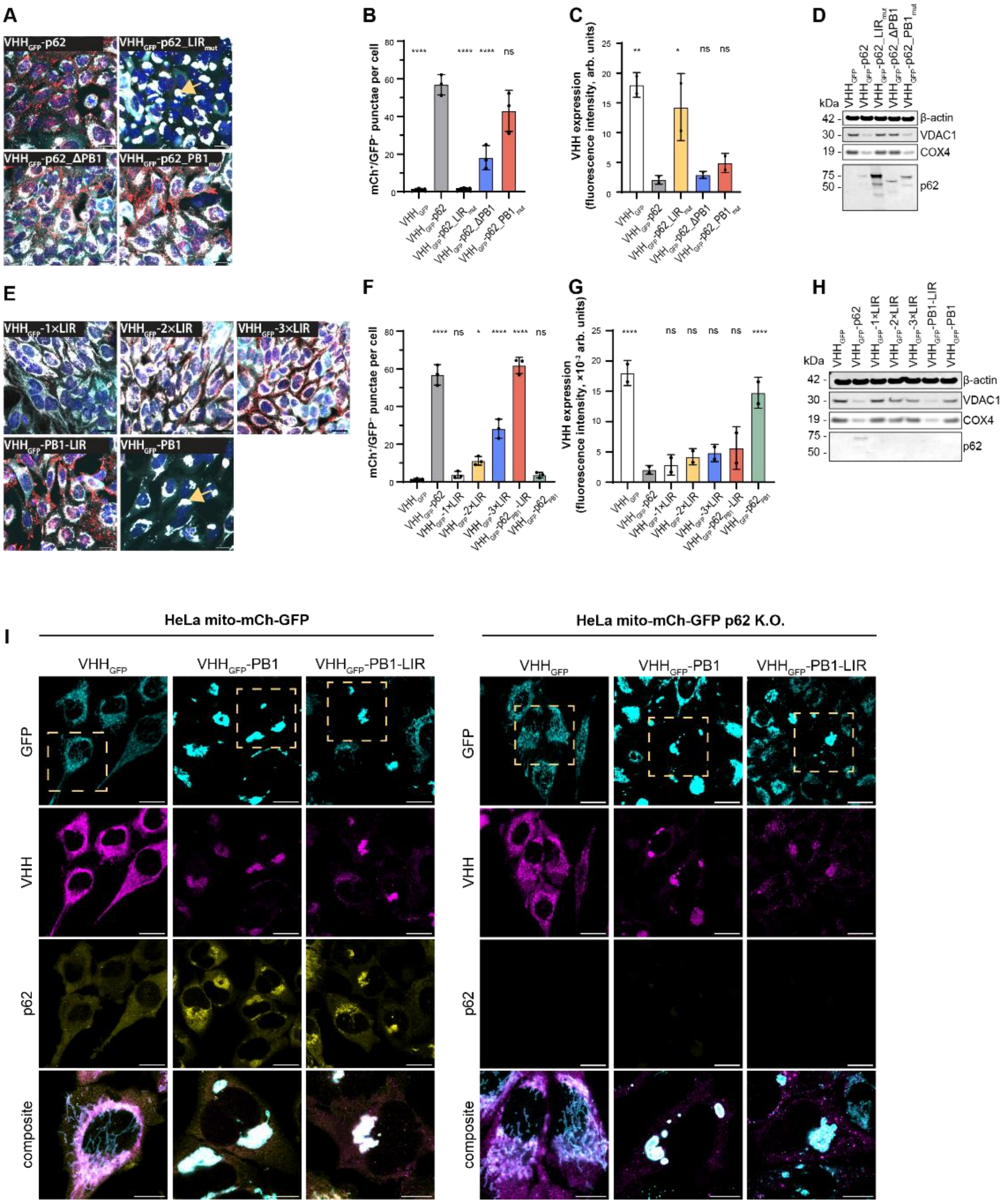

Figure S6. Evaluation of p62 mutants, and LIR- and PB1 containing constructs in p62 K.O. cells.

(A) Representative confocal fluorescence microscopy images showing stable HeLa mito-mCh-GFP p62 K.O. cells with inducible expression of fusions of VHH_{GFP} and p62 mutants for 72 h. Red = mCh, cyan = GFP, blue = nuclei. Scale bars are 30 µm. Large mCh^+/GFP^+ structures are indicated by yellow arrows.

(B) Quantification of mCh^+/GFP^- punctae of panel A.

(C) Expression levels of VHH_{GFP} fusions of panel A. Fluorescence intensities were assessed by immunofluorescence microscopy.

(D) Immunoblotting of stable HeLa mito-mCh-GFP p62 K.O. cells with inducible expression of fusions of VHH_{GFP} and p62 mutants for 72 h.

(E) Representative confocal fluorescence microscopy images showing stable HeLa mito-mCh-GFP p62 K.O. cells with inducible expression of fusions of VHH_{GFP} and LIR- and PB1 containing constructs for 72 h Red = mCh, cyan = GFP, blue = nuclei. Scale bars are 30 µm. Large mCh^+/GFP^+ structures are indicated by yellow arrows.

(F) Quantification of mCh^+/GFP^- punctae of panel E.

(G) Expression levels of VHH_{GFP} fusions of panel E. Fluorescence intensities were assessed by immunofluorescence microscopy.

(H) Immunoblotting of stable HeLa mito-mCh-GFP p62 K.O. cells with inducible expression of fusions of VHH_{GFP} and LIR- and PB1 containing constructs for 72 h.

(I) Representative immunofluorescence Airyscan images of stable HeLa mito-mCh-GFP and HeLa mito-mCh-GFP p62 K.O. cells with inducible expression of fusions of VHH_{GFP} and p62, PB1 and PB1-LIR for 72 h. Cells were fixed and immunostained with anti-p62 and anti-VHH. Cyan = GFP, magenta = VHH, yellow = p62. Scale bars are 20 µm and 5 µm (magnification).

Data are shown as mean and standard deviation from *n* = 2 (panel C and G) and *n=3* (panel B and F) independent biological replicates. Statistical analysis was performed using an ordinary one-way ANOVA with multiple comparison of each data point against VHH_{GFP}-p62 (panels B and C) or VHH_{GFP} (panels F and G). *P*-value summary: ns = ($P > 0.05$); * = ($P \leq 0.05$); ** = ($P \leq 0.01$); *** = ($P \leq 0.001$); **** = ($P \leq 0.0001$). Exact *p*-values are shown in the source data.

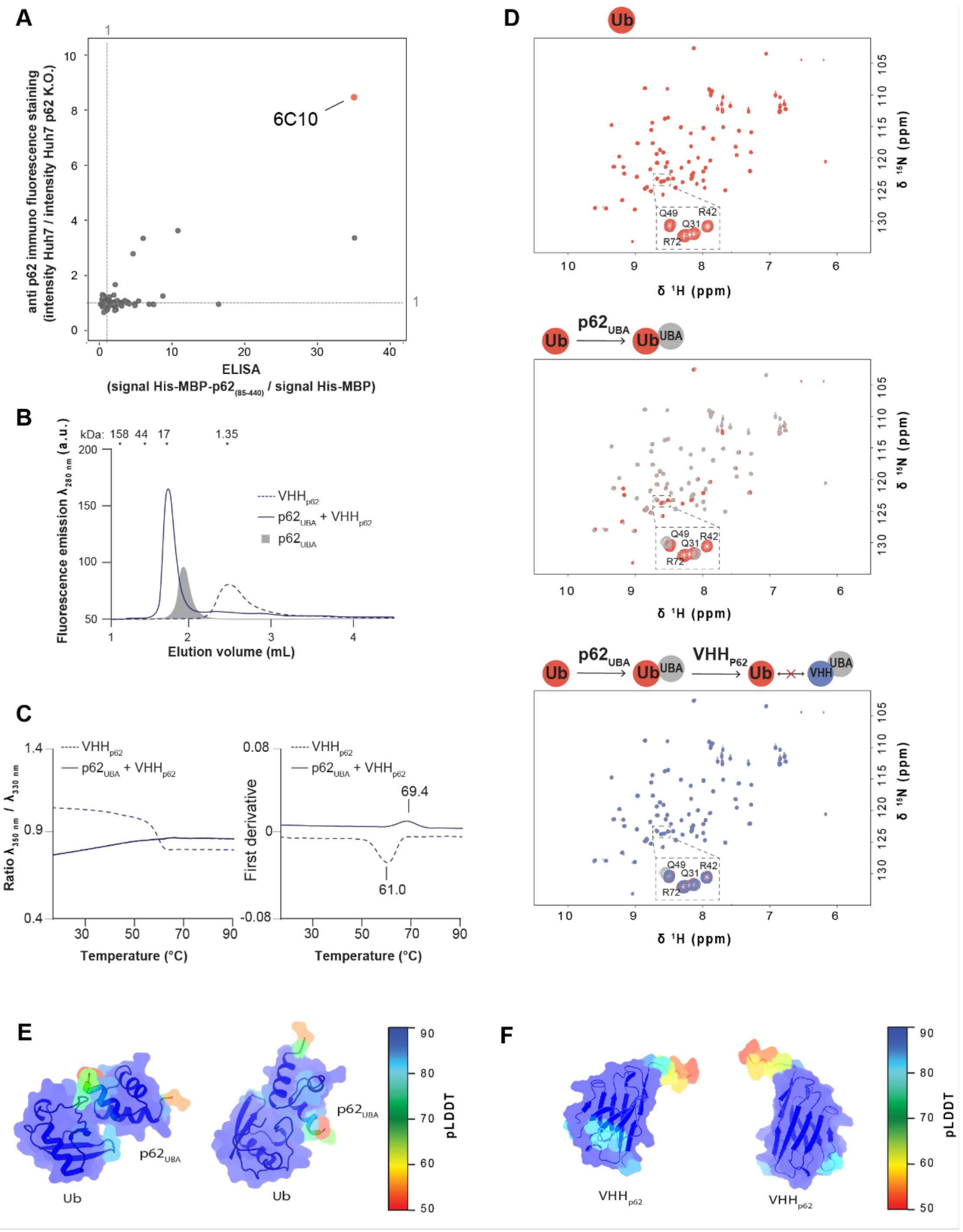

Figure S7. Generation and analytical QC of VHH$_{p62}$.

(A) Selection of the anti-p62 VHH with the lowest signal to noise ratio using immunofluorescence microscopy and ELISA assays. Immunofluorescence microscopy experiments were performed using periplasmic extracts of *E. coli* expressing VHHs followed by staining against VHH. The ratio of the fluorescence intensities obtained in Huh7 and Huh7 p62. K.O. was then calculated. ELISA assays were performed using periplasmic extracts of *E. coli* expressing VHHs. The ratio was calculated from the signal obtained from plates coated with the full antigen His-MBP-p62$_{(85\text{-}440)}$ or the control His-MBP.

(B) Size exclusion chromatography (SEC) of recombinant VHH$_{p62}$, p62$_{UBA}$, and a 1:1.5 mixture of VHH$_{p62}$ and p62$_{UBA}$.

(C) Nano differential scanning fluorimetry of VHH$_{p62}$ alone and a 1:2 mixture of VHH$_{p62}$ and p62$_{UBA}$.

(D) 2D projection of a (^{1}H,^{15}N)-SOFAST-HSQC spectra of ^{13}C,^{15}N-labeled ubiquitin, a 1:1 mixture of ^{13}C,^{15}N-labeled ubiquitin and p62$_{UBA}$, and a 1:1:1.2 mixture of ^{13}C,^{15}N-labeled ubiquitin, p62$_{UBA}$, and VHH$_{p62}$.

(E) Alphafold-Multimer prediction of the p62$_{UBA}$:ubiquitin complex with pLDDT overlay viewed from two different angles.

(F) Alphafold2 prediction of VHHp62 used for refinement of the X-Ray crystallography structure.

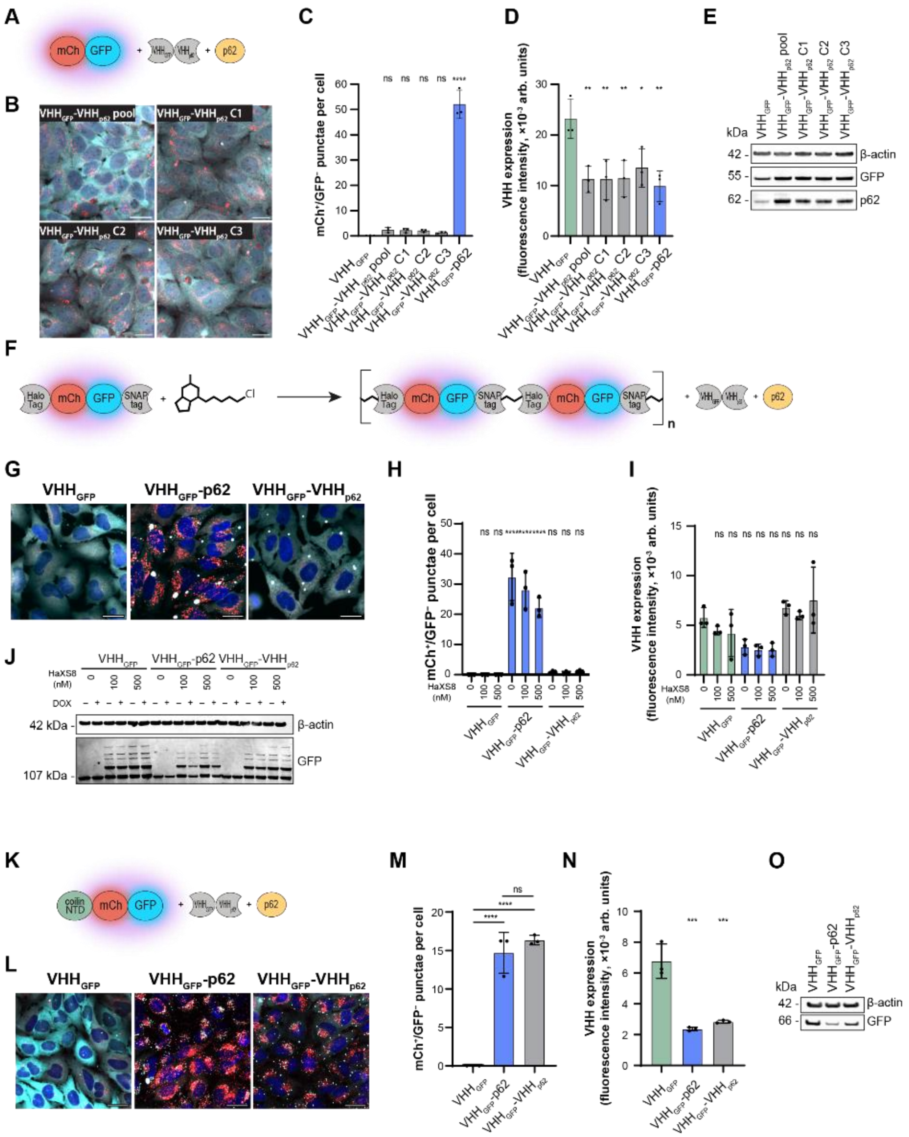

Figure S8. Effect of VHH_{GFP}-VHH_{p62} mediated induced proximity of various cytosolic targets with p62.

(A) Schematic representation of the approach used to induce VHH_{GFP}-VHH_{p62} dependent proximity between the cytosolic target cyt-mCh-GFP and p62.
(B) Representative confocal fluorescence microscopy image showing stable HeLa cyt-mCh-GFP cells with inducible expression of VHH_{GFP}-VHH_{p62} for 72 h. Red = mCh, cyan = GFP, blue = nuclei. Scale bars are 30 μm.
(C) Quantification of mCh^+/GFP^- punctae of panel B.
(D) Expression levels of VHH of panel B. Fluorescence intensities were assessed by immunofluorescence microscopy.
(E) Immunoblotting of stable HeLa cyt-mCh-GFP cells with inducible expression of fusions of VHH_{GFP}-VHH_{p62} for 72 h.
(F) Schematic representation of the approach used to oligomerize the cytosolic target HaloTag-mCh-GFP-SNAPtag and induce VHH_{GFP}-VHH_{p62} dependent proximity with p62.
(G) Representative confocal fluorescence microscopy image showing stable HeLa HaloTag-mCh-GFP-SNAPtag cells treated with HaXS8 (500 nM) expressing VHH_{GFP}, VHH_{GFP}-p62, or VHH_{GFP}-VHH_{p62} for 72 h. Red = mCh, cyan = GFP, blue = nuclei. Scale bars are 30 μm
(H) Quantification of mCh^+/GFP^- punctae of panel G.
(I) Expression levels of VHH of panel G. Fluorescence intensities were assessed by immunofluorescence microscopy.
(J) Immunoblotting of stable HeLa HaloTag-mCh-GFP-SNAPtag cells treated with HaXS8 (0 nM, 100 nM ,or 500 nM) expressing VHH_{GFP}, VHH_{GFP}-p62, or VHH_{GFP}-VHH_{p62} for 72 h.
(K) Schematic representation of the approach used to induce VHH_{GFP}-VHH_{p62} dependent proximity between the oligomeric, cytosolic target $coilin_{NTD}$-mCh-GFP and p62.
(L) Representative confocal fluorescence microscopy image showing stable HeLa $coilin_{NTD}$-mCh-GFP cells with inducible expression of VHH_{GFP}, VHH_{GFP}-p62, or VHH_{GFP}-VHH_{p62} for 72 h. Red = mCh, cyan = GFP, blue = nuclei. Scale bars are 30 μm.
(M) Quantification of mCh^+/GFP^- punctae of panel L.
(N) Expression levels of VHH of panel L. Fluorescence intensities were assessed by immunofluorescence microscopy.
(O) Immunoblotting of stable HeLa $coilin_{NTD}$-mCh-GFP cells with inducible expression of VHH_{GFP}, VHH_{GFP}-p62, or VHH_{GFP}-VHH_{p62} for 72 h.

Data are shown as mean and standard deviation from n = 3 independent biological replicates. Statistical analysis was performed using an ordinary one-way ANOVA with multiple comparison of each data point against VHH_{GFP} or using an unpaired, two-sided t-test performed on VHH_{GFP} versus VHH_{GFP}-VHH_{p62} (panel M). P-value summary: ns = ($P > 0.05$); * = ($P \leq 0.05$); ** = ($P \leq 0.01$); *** = ($P \leq 0.001$); **** = ($P \leq 0.0001$). Exact p-values are shown in the source data.

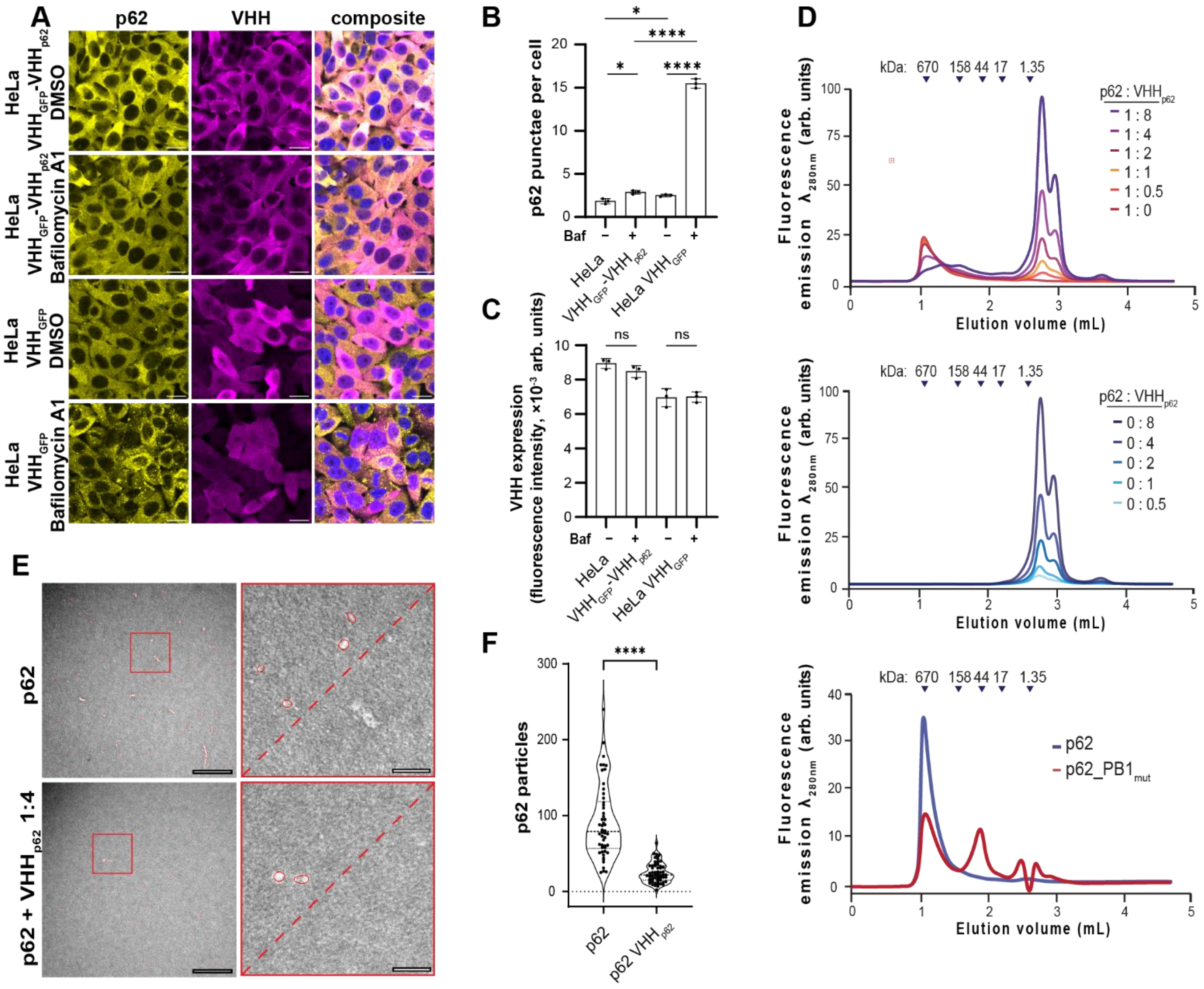

Figure S9. Binding of VHH_{p62} to p62 inhibits p62 self-oligomerization.

(A) Representative images of stable HeLa cells expressing VHH_{GFP} or VHH_{GFP}-VHH_{p62} for 24 h and treated with DMSO or Bafilomycin A1 (100 nM) for 4 h. Cells were fixed and immunostained. yellow = p62, magenta = VHH. Scale bars are 20 µm.

(B) Quantification of p62 punctae panel A.

(C) Expression levels of VHH in panel A. Expression levels were assessed by immunofluorescence microscopy.

(D) SEC analysis of increasing concentrations of recombinant VHH_{p62},in the presence or absence of recombinant full length p62 as well as p62_$PB1_{mut}$.

(E) Representative negative staining transmission electron microscopy images of recombinant full length p62 with and without addition of recombinant VHH_{p62}. Scale bars are 200 and 50 nm (magnification).

(F) Quantification of high-molecular weight particles formed by p62, displayed in panel F.

Data are shown as mean and standard deviation from *n* = 3 independent biological replicates (panels B and C) or *n* = 3 technical replicates (panel F). Statistical analysis was performed using an unpaired, two-sided *t*-tests. *P*-value summary: ns = ($P > 0.05$); * = ($P \leq 0.05$); ** = ($P \leq 0.01$); *** = ($P \leq 0.001$); **** = ($P \leq 0.0001$). Exact *p*-values are shown in the source data.

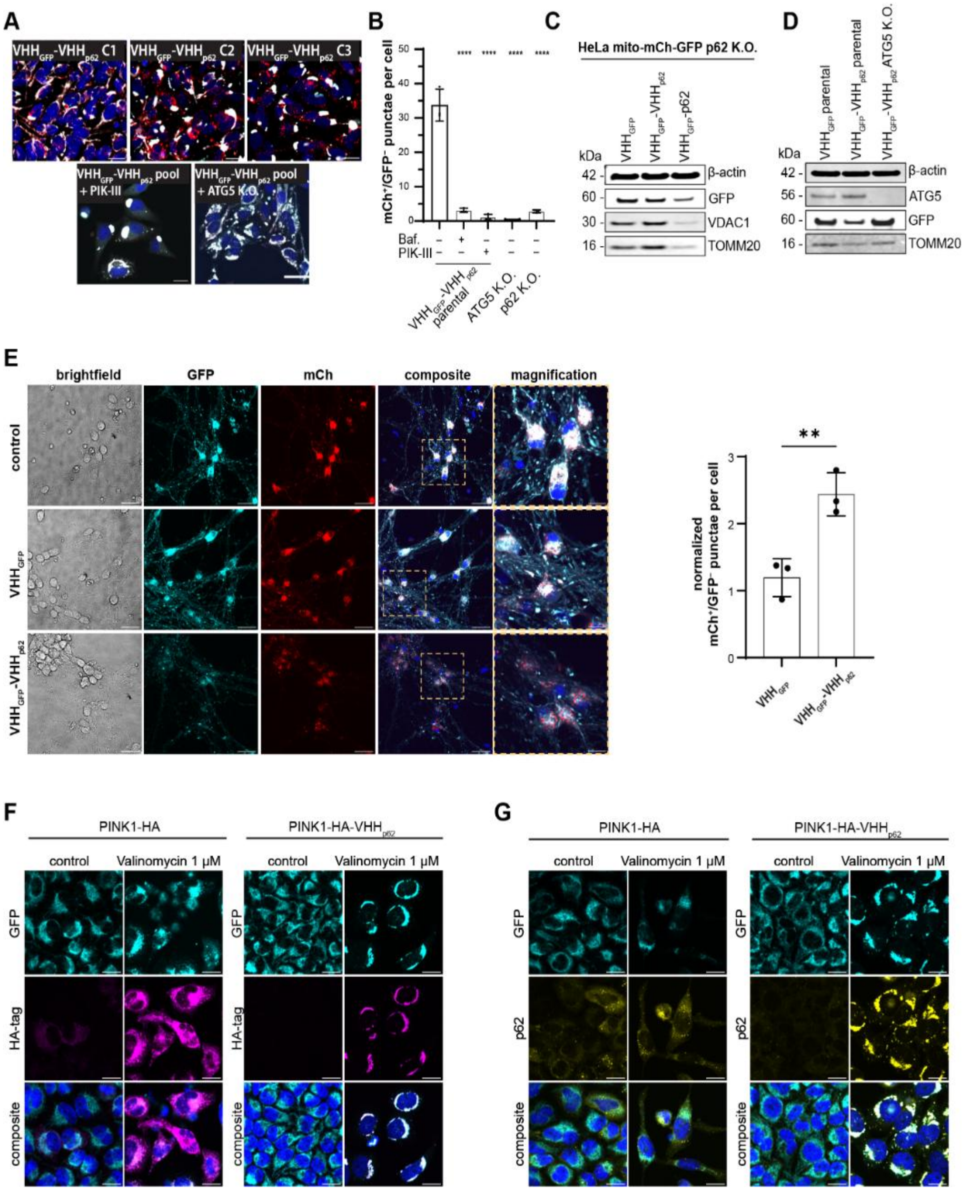

Figure S10. Targeted degradation of mitochondria using VHH$_{GFP}$-VHH$_{p62}$ and PINK1-HA-VHH$_{p62}$

(A) Representative confocal fluorescence microscopy image showing stable HeLa mito-mCh-GFP cells with inducible expression of VHH$_{GFP}$-VHH$_{p62}$ for 72 h. Red = mCh, cyan = GFP, blue = nuclei. Scale bars are 30 µm.

(B) Quantification of mCh$^+$/GFP$^-$ punctae in stable HeLa mito-mCh-GFP cells with inducible expression of VHH$_{GFP}$-VHH$_{p62}$ for 72 h that were treated with PIK-III (5 µM) for 72 h or Bafilomycin A1 (100 nM) for 15 h prior to imaging as well as stable HeLa mito-mCh-GFP ATG5 K.O and stable HeLa mito-mCh-GFP p62 K.O cells with inducible expression of VHH$_{GFP}$-VHH$_{p62}$ for 72 h.

(C) Immunoblot of stable HeLa mito-mCh-GFP p62 K.O. cells with inducible expression of VHH$_{GFP}$, VHH$_{GFP}$-p62, or VHH$_{GFP}$-VHH$_{p62}$ for 72 h.

(D) Immunoblot of stable HeLa mito-mCh-GFP and stable HeLa mito-mCh-GFP ATG5 K.O. cells with inducible expression of VHH$_{GFP}$-VHH$_{p62}$ for 72 h.

(E) Representative confocal fluorescence microscopy images and quantification of mCh$^+$/GFP$^-$ punctae in live iPSC-derived NGN2 neurons expressing mito-mCh-GFP and VHH$_{GFP}$, VHH$_{GFP}$-VHH$_{p62}$ for 7 days. Red = mCh, cyan = GFP, blue = nuclei. Scale bars are 30 µm.

(F) Representative confocal immunofluorescence images of stable HeLa mito-mCh-GFP cells with constitutive expression of PINK1-HA or PINK1-HA-VHH$_{p62}$ with and without treatment of Valinomycin (0.2 µM and 1 µM) for 4 h before washing and incubation for 72 h. Cells were fixed and immunostained with anti-HA-tag. Cyan = GFP, magenta = HA-tag, blue = nuclei. Scale bars are 30 µm.

(G) Representative confocal immunofluorescence images of stable HeLa mito-mCh-GFP cells with constitutive expression of PINK1-HA or PINK1-HA-VHH$_{p62}$ with and without treatment of Valinomycin (0.2 µM and 1 µM) for 4 h before washing and incubation for 72 h. Cells were fixed and immunostained with anti-p62. Cyan = GFP, yellow = p62, blue = nuclei. Scale bars are 30 µm.

Data are shown as mean and standard deviation from n = 3 independent biological replicates. Statistical analysis was performed using an ordinary one-way ANOVA with multiple comparison of each data point against untreated, parental VHH$_{GFP}$-VHH$_{p62}$ (panel B) or using an unpaired, two-sided t-test performed on VHH$_{GFP}$ versus VHH$_{GFP}$-VHH$_{p62}$ (panel E). P-value summary: ns = (P > 0.05); * = ($P \leq 0.05$); ** = ($P \leq 0.01$); *** = ($P \leq 0.001$); **** = ($P \leq 0.0001$). Exact p-values are shown in the source data.
