# Supplementary material for: Development of a p62 biodegrader for autophagy targeted degradation
Source: Nat Commun. 2025 Dec 3;16:10858. doi: 10.1038/s41467-025-65868-9 (PMC12675543; doi:10.1038/s41467-025-65868-9)
Supplement: Supplementary file 2 — Description of Additional Supplementary Files [file 41467_2025_65868_MOESM2_ESM.pdf]

Description of Supplementary Files

Supplementary Dataset 1

Description: Results table from the induced proximity cDNA screen presented in Figure 1B.

Supplementary Dataset 2

Description: Affinity proteomics of VHHp62 binding partners in Huh7 and Huh7 p62 K.O. cells shown in Figure 4G.

Supplementary Dataset 3

Description: Sequences of constructs used in this study.
