# Supplementary material for: Development of a p62 biodegrader for autophagy targeted degradation
Source: Nat Commun. 2025 Dec 3;16:10858. doi: 10.1038/s41467-025-65868-9 (PMC12675543; doi:10.1038/s41467-025-65868-9)
Supplement: Supplementary file 6 — Reporting Summary [file 41467_2025_65868_MOESM6_ESM.pdf]

nature portfolio

Corresponding author(s): Beat Nyfeler, Zacharias Thiel

Last updated by author(s): 09/09/2025

Reporting Summary

Nature Portfolio wishes to improve the reproducibility of the work that we publish. This form provides structure for consistency and transparency in reporting. For further information on Nature Portfolio policies, see our Editorial Policies and the Editorial Policy Checklist.

Statistics

For all statistical analyses, confirm that the following items are present in the figure legend, table legend, main text, or Methods section.

n/a	Confirmed	
☐	☒	The exact sample size (*n*) for each experimental group/condition, given as a discrete number and unit of measurement
☐	☒	A statement on whether measurements were taken from distinct samples or whether the same sample was measured repeatedly
☐	☒	The statistical test(s) used AND whether they are one- or two-sided<br>*Only common tests should be described solely by name; describe more complex techniques in the Methods section.*
☒	☐	A description of all covariates tested
☒	☐	A description of any assumptions or corrections, such as tests of normality and adjustment for multiple comparisons
☐	☒	A full description of the statistical parameters including central tendency (e.g. means) or other basic estimates (e.g. regression coefficient) AND variation (e.g. standard deviation) or associated estimates of uncertainty (e.g. confidence intervals)
☐	☒	For null hypothesis testing, the test statistic (e.g. *F*, *t*, *r*) with confidence intervals, effect sizes, degrees of freedom and *P* value noted<br>*Give P values as exact values whenever suitable.*
☒	☐	For Bayesian analysis, information on the choice of priors and Markov chain Monte Carlo settings
☒	☐	For hierarchical and complex designs, identification of the appropriate level for tests and full reporting of outcomes
☒	☐	Estimates of effect sizes (e.g. Cohen's *d*, Pearson's *r*), indicating how they were calculated

Our web collection on statistics for biologists contains articles on many of the points above.

Software and code

Policy information about availability of computer code

Data collection	Imaging: Zen 3.2 blue edition (Zeiss), IN Cell Analyzer 2500 v 7.2 (GE Healthcare), CellVoyager (Yokogawa)<br>Western: LI-COR Acquisition Software 1.0<br>SPR: Biacore T-200 (GE Healthcare)<br>EM: Thermofisher Scientific Tecnai Spirit
Data analysis	Imaging: ImageJ v. 1.53t, CellProfiler v. 4.2.4.<br>Data formatting, plotting, and statistics: KNIME v. 4.6.5, GraphPad Prism v. 10.1.2, TIBCO Spotfire Analyst v.12.0.9<br>Proteomics: MaxQuant v. 2.2.0<br>NMR: Bruker TopSppin 3.2.1<br>Crystallization: autoPROC v.1.1.7, Aimless v. 0.7.7, Alphafold2, Phenix process_predicted_model v1.20.4459, Phase v. 2.8.3, Coot v. 0.9.8.7, CCP4I2 v.9.0.011, PyMOL v. 2.1.0<br>SPR: Biacore Evaluation Software (GE Healthcare)

For manuscripts utilizing custom algorithms or software that are central to the research but not yet described in published literature, software must be made available to editors and reviewers. We strongly encourage code deposition in a community repository (e.g. GitHub). See the Nature Portfolio guidelines for submitting code & software for further information.

Data

Policy information about availability of data

All manuscripts must include a data availability statement. This statement should provide the following information, where applicable:

- Accession codes, unique identifiers, or web links for publicly available datasets
- A description of any restrictions on data availability
- For clinical datasets or third party data, please ensure that the statement adheres to our policy

All data are in the main text, the supplementary materials, or in the source data file. Crystallography data are deposited in the RCSB protein data bank (PDB 9H1J). The mass spectrometry proteomics data have been deposited to the ProteomeXchange Consortium via the PRIDE partner repository with the dataset identifier PXD057810.

Research involving human participants, their data, or biological material

Policy information about studies with human participants or human data. See also policy information about sex, gender (identity/presentation), and sexual orientation and race, ethnicity and racism.

Reporting on sex and gender	not applicable
Reporting on race, ethnicity, or other socially relevant groupings	not applicable
Population characteristics	not applicable
Recruitment	not applicable
Ethics oversight	not applicable

Note that full information on the approval of the study protocol must also be provided in the manuscript.

Field-specific reporting

Please select the one below that is the best fit for your research. If you are not sure, read the appropriate sections before making your selection.

☒ Life sciences ☐ Behavioural & social sciences ☐ Ecological, evolutionary & environmental sciences

For a reference copy of the document with all sections, see nature.com/documents/nr-reporting-summary-flat.pdf

Life sciences study design

All studies must disclose on these points even when the disclosure is negative.

Sample size	Sample sizes were chosen based on previous publications, which were sufficient for statistical analysis.
Data exclusions	No data were excluded from the analysis.
Replication	All attempts of replication were successful. The replication numbers were described in the corresponding figure legends.
Randomization	not applicable
Blinding	not applicable

Reporting for specific materials, systems and methods

We require information from authors about some types of materials, experimental systems and methods used in many studies. Here, indicate whether each material, system or method listed is relevant to your study. If you are not sure if a list item applies to your research, read the appropriate section before selecting a response.

Materials & experimental systems

n/a	Involved in the study	
☐	☒	Antibodies
☐	☒	Eukaryotic cell lines
☒	☐	Palaeontology and archaeology
☐	☒	Animals and other organisms
☒	☐	Clinical data
☒	☐	Dual use research of concern
☒	☐	Plants

Methods

n/a	Involved in the study	
☒	☐	ChIP-seq
☒	☐	Flow cytometry
☒	☐	MRI-based neuroimaging

Antibodies

Antibodies used	The anti-COX4 (ab14744, 1:400 for WB), anti-mCherry (ab125096, 1:500 for WB), anti-TOMM20 (ab283317, 1:500 for WB) and anti-LAMP1 (ab25630-100, 1:200 for IF) antibodies were purchased from Abcam. The anti-p62 (610833, 1:500 for WB and IF) antibody was purchased from BD Biosciences. The anti-β-actin (3700S, 1:5000 for WB), anti-GFP (2956S, 1:500 for WB), and anti-VDAC1 (4866S, 1:500 for WB) antibodies were purchased from Cell Signaling Technology. The anti-VHH-ifluor647 conjugate (A02170-200, 1:500 for IF) antibody was purchased from Genescript. The anti-VHH (200-401-GM6, 1:500 for WB) antibody was purchased from Rockland. The anti-HaloTag antibody, (G921A, 1:500 for WB) was purchased from Promega. The Alexa Fluor™ 488 Goat anti-Mouse IgG (A11029, 1:1000 for IF), Alexa Fluor™ 647 Donkey anti-Mouse IgG (A31571, 1:1000 for IF), Alexa Fluor™ 488 Goat anti-Rabbit IgG (A11008, 1:1000 for IF) and Alexa Fluor™ 405 Goat anti-Mouse IgG (A31553, 1:500 for IF) antibodies were purchased from Invitrogen. The IRDye® 800CW Donkey anti-Rabbit IgG (926-32213, 1:5000 for WB) and IRDye® 680RD Donkey anti-Mouse IgG (926-68072, 1:5000 for WB) antibodies were purchased from LICORbio.
Validation	The anti-p62 (610833, BD Biosciences) was validated using HuH7 p62 K.O. cells. All other antibodies were validated by the manufacturers.

Eukaryotic cell lines

Policy information about cell lines and Sex and Gender in Research

Cell line source(s)	HeLa (ATCC, CRM-CCL-2), HuH-7 (JCRB, JCRB0403), human dermal fibroblasts (Invitrogen, C-013-5C)
Authentication	The cell lines were authenticated by the suppliers.
Mycoplasma contamination	Routine testing for mycoplasma was performed and was negative for all tested cell lines
Commonly misidentified lines (See ICLAC register)	not applicable

Animals and other research organisms

Policy information about studies involving animals; ARRIVE guidelines recommended for reporting animal research, and Sex and Gender in Research

Laboratory animals	Llama
Wild animals	not applicable
Reporting on sex	not applicable
Field-collected samples	not applicable
Ethics oversight	not applicable

Note that full information on the approval of the study protocol must also be provided in the manuscript.

Plants

Seed stocks	not applicable
Novel plant genotypes	not applicable
Authentication	not applicable
